# A multi-jointed underactuated robot hand with fluid-driven stretchable tubes

**DOI:** 10.1186/s40638-018-0086-6

**Published:** 2018-06-20

**Authors:** Yuangen Wei, Yini Ma, Wenzeng Zhang

**Affiliations:** 1 Department of Mechanical Engineering, Tsinghua University, Beijing, 100084 China; 2 Department of Physics, Tsinghua University, Beijing, 100084 China

**Keywords:** Multi-fingered robot hand, Self-adaptive mechanism, Artificial muscle, Biologically inspired robotics

## Abstract

Inspired from flexible bending of octopus’ tentacles and outside-driving kind of traditional exoskeletons, this paper proposed a novel self-adaptive underactuated finger mechanism, called OS finger. OS finger is similar to an octopus’ tentacle and consists of an artificial muscle which is through all joints and driven by fluid, eight serial-hinged joints, and force-changeable assembly. The force-changeable assembly is mainly composed of a spring and elastic rubber membrane, which is coordinated for stable grasping by a layer of rubber material in the surface of the finger. OS finger can execute different grasping modes depending on the shapes and dimensions of the grasped objects and grip objects in a gentle and form-fitting manner. The OS finger combines good qualities of both rigid grasp of traditional fingers and form-fitting grasp of flexible fingers. Kinematic analysis and experimental results show that the OS robot Hand with four OS fingers is valid for precise pinching, self-adaptive powerful encompassing, and grasping forces that are freely changeable in a wide range. With the advantage of high self-adaptation, various grasp configurations and large range of grasping forces, the OS Hand has a wide range of applications in the area of service robotics which requires a lot of flexible operations of general grasping, moving and releasing.

## Background

In recent years, breathtaking developments in robotics have been witnessed all over the world. The field of robotic hands including dexterous hands and underactuated hands is emphasized and developed.

Over the past 30 years, researchers have made plentiful achievements on the study of dexterous hand. For instance, Stanford/JPL dexterous hand was designed and analyzed by Salisbury et al. [1], which has three 3-DOF fingers actuated by 12 DC motors; each joint of this hand can be flexed and extended independently by one actuator. Gifu II hand, designed by Kawasaki et al. [2], has 5 fingers whose all joints are actuated by servomotors, which can perform dexterous object manipulation like the human hand; Utah/MIT dexterous hand was designed by Jacobsen et al. [3], which has four 4-DOF fingers with 32 independent tendons and 32 pneumatic cylinders [4]; the hand can be used as a high flexible tool for the study of machine dexterity. Dexterous hand can do almost all the movements and gestures of human hand. In fact, almost each DOF of a dexterous hand needs an actuator to drive, which makes the hand high dependence on control and high cost on manufacturing and using expenses.

On the contrary, underactuated hands use fewer motors to drive more DOFs, and the underactuated hands have a very amazing feature: self-adaptation in grasping, which let the hands easy to control. Many studies have done in the field of underactuated hands: Birglen et al. [5–7] designed many kinds of underactuated grippers and gave force analyses on them. Dollar et al. [8, 9] gave a SDM robust robotic grasper which uses a single actuator to actuate 8 DOFs; Tan et al. [10] designed a multi-fingered hand using hydraulic actuation with fluidic actuators; the hand has 14 DOFs which can bend when hydraulic pressure is applied by a water pump. Underactuated hand does not have complex sensor, algorithm and control systems. But the insufficiency of underactuated hand is that the contact points on objects are too narrow to protect the grasped objects.

To make up the shortcoming of the hands mentioned above, some flexible robot hands have earned widespread respect and have been widely researched [11]. Excellent examples are the Multi-Choice Gripper [12], the Flexible Shape Gripper [13], the soft tentacles [14] and so on. However, the grasping force of these hands is too small to grasp a heavier object.

We find that some underactuated hand exoskeletons have developed [15, 16]. Transmission mechanisms of these hand exoskeletons are designed outside the joints. And the underactuated mechanisms are significant for simplifying control systems. These hand exoskeletons can get much more powerful grasping force due to the bigger outside-driving space. Also, we learn that more joints can better adapt to objects of different shapes and sizes [17]. Inspired by all of that and the flexible bending of octopus’ tentacles, this paper proposes a novel self-adaptive underactuated multi-fingered hand (OS Hand), combing the advantages of traditional rigid robot hands and flexible robot hands. OS Hand has high adaptability and powerful grasping force which can be widely used in industrial fields.

## Design of the OS Hand

In this section, the principle of OS finger (called Artificial Tentacle) is presented firstly. Secondly, the working process of the Artificial Tentacle is presented. Finally, compositions of OS Hand are introduced.

### Principle of artificial tentacle

**Fig. 1:**
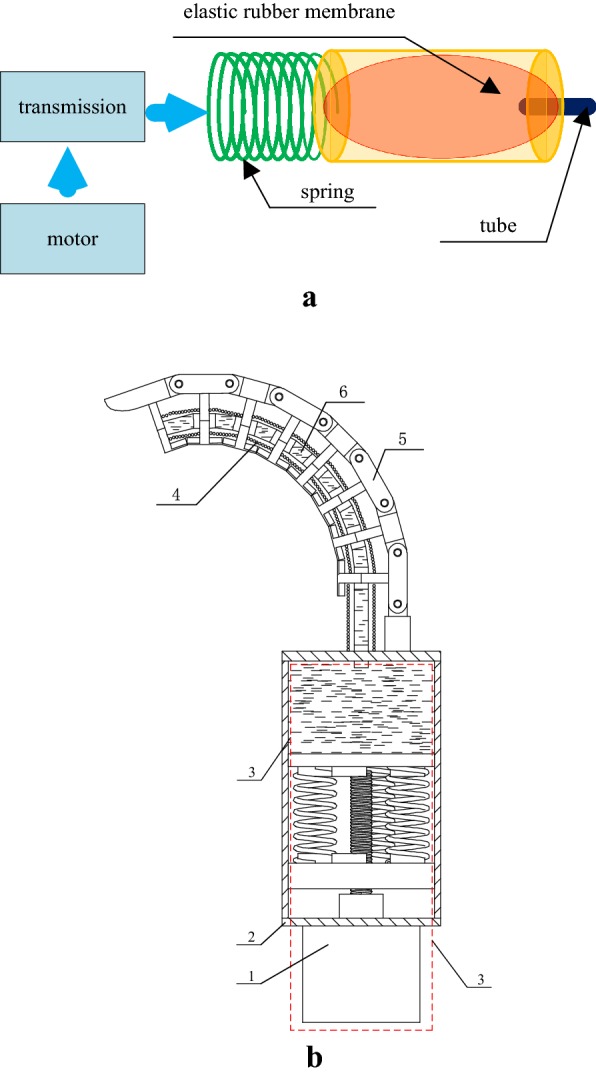
Principle of artificial tentacle. 1—motor; 2—base; 3—force-changeable assembly; 4—artificial muscle; 5—eight serial-hinged joints; 6—fluid. **a** Principle of force-changeable assembly. **b** Artificial tentacle

The artificial tentacle consists of an artificial muscle, eight serial-hinged joints and force-changeable assembly, as shown in Fig. 1. The artificial muscle is composed of a stretchable flexible tube which goes through all joints and is driven by fluid. The force-changeable assembly is mainly composed of a spring and elastic rubber membrane, which is coordinated for stable grasping by a layer of rubber material in the surface of the tentacle. There is fluid in the elastic rubber membrane. The joints of the eight serial-hinged joints are made of rigid links, providing support for artificial tentacles and improving the grasping strength. The strength of joints and stretchable flexible tube are adequate to sustain heavy objects.

### Grasping process of artificial tentacle

**Fig. 2:**
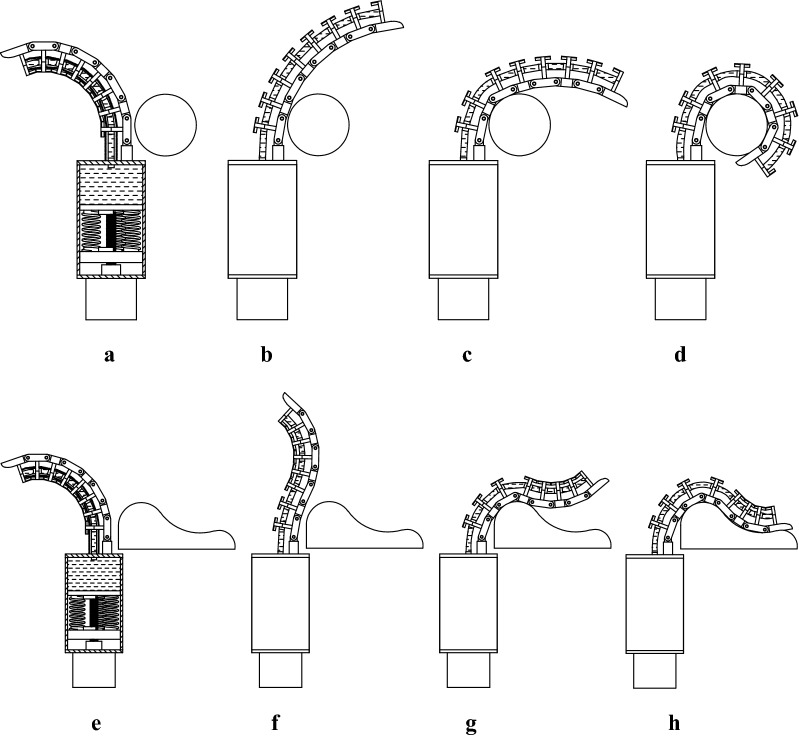
Grasping process of artificial tentacle. **a** Grasping step 1 of regular object. **b** Grasping step 2 of regular object. **c** Grasping step 3 of regular object. **d** Grasping step 4 of regular object. **e** Grasping step 1 of irregular object. **f** Grasping step 2 of irregular object. **g** Grasping step 3 of irregular object. **h** Grasping step 4 of irregular object

Grasping process of artificial tentacle is shown in Fig. 2. The initial position of the tentacle is shown in Fig. 2a, e, where the artificial muscle is in a contracted state.

When the motor runs forward, the fluid is pushed into the artificial muscle. Then, the artificial muscle elongates, and the artificial tentacle rotates toward object. When the motor continues to run, the fluid is continuously pushed into the artificial muscle, and the artificial muscle constantly elongates. Each joint rotates separately until it meets the object and then stops. When all joints meet the object, the grasping process finishes, as is shown in Fig. 2.

When the motor continues to run, the force of the spring changes and the artificial tentacle can get different grasping forces.

For different shapes and sizes of objects, the artificial tentacle has a good self-adaptability and can get a very reliable grasping strength, as is shown in Fig. 2d, h.

### Components of OS Hand

**Fig. 3:**
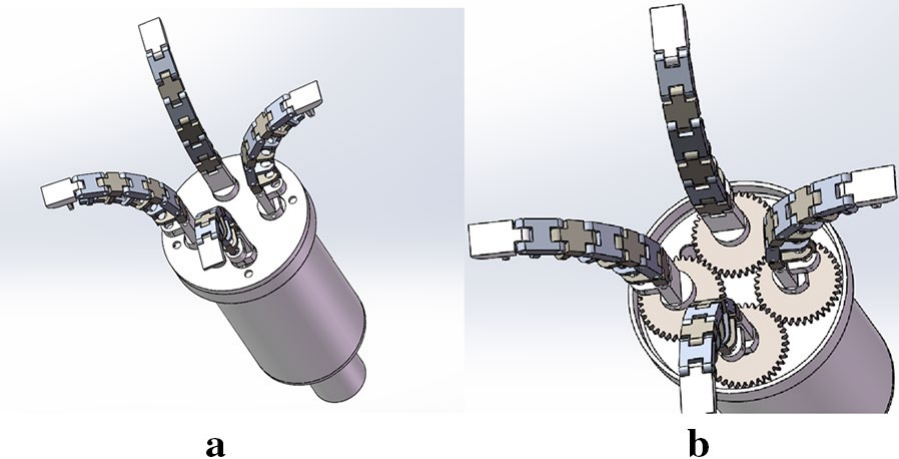
Components of OS Hand. **a** Virtual model of OS Hand. **b** Perspective of the virtual model

As is shown in Fig. 3, OS Hand has four artificial tentacles. All the artificial tentacles are the same. And the four artificial muscles of the four artificial tentacles, i.e., the four stretchable flexible tubes, are connected. That’s to say, the liquid distribution pipes are connected. So the four artificial tentacles are driven by only one motor concurrently. And that’s of great significance to simplify the driving system as well as the control system and can help to reduce costs. What’s more, each artificial tentacle is connected with a gear, and the four gears are meshed together, as is shown in Fig. 3b. And one of the four gears is connected with a motor, so the pose of the tentacles can change by driving the motor. And that make it possible for OS Hand to execute variety of grasping modes, and so the practicality of OS Hand can be increased.

## Analysis Methods

### Kinematics analysis

OS Hand has a wide range of applications. In order to confirm the position and posture of the tentacles, we employed the D–H method to perform the kinematics analysis.

**Fig. 4:**
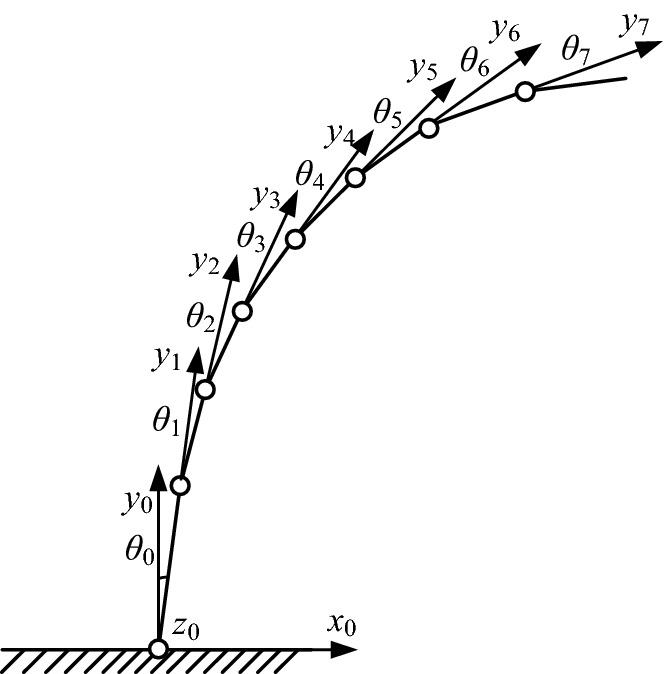
Kinematic analysis of single tentacle

Since the four tentacles in our experiment are all the same, we focus on only one tentacle. The kinematic analysis of single tentacle is shown in Fig. 4.

During our analysis, $x_{0} y_{0} z_{0}$ is a static world coordinate system and others are joint coordinate systems. Considering the size of the finger and manufacturability of the parts, we set the D–H parameters as Table 1.

**Table 1:** D–H parameters

Joint *i*	*α*_*i*_	*l*_*i*_	*d*_*i*_	*θ*_*i*_	Range of *θ*_*i*_/°
0	0	*l*_0_	0	*θ*_0_	− 60 to 90
1	0	*l*_1_	0	*θ*_1_	0–90
2	0	*l*_2_	0	*θ*_2_	0–90
…	…	…	…	…	…
6	0	*l*_6_	0	*θ*_6_	0–90
7	0	*l*_7_	0	*θ*_7_	0–90

The interpretations of the quantities are as follows:

- $\alpha_{i}$: angle of two adjacent joint axes.
- $l_{i}$: length of joint.
- $d_{i}$: distance between joints.
- $\theta_{i}$: angle of two adjacent joints.

**Fig. 5:**
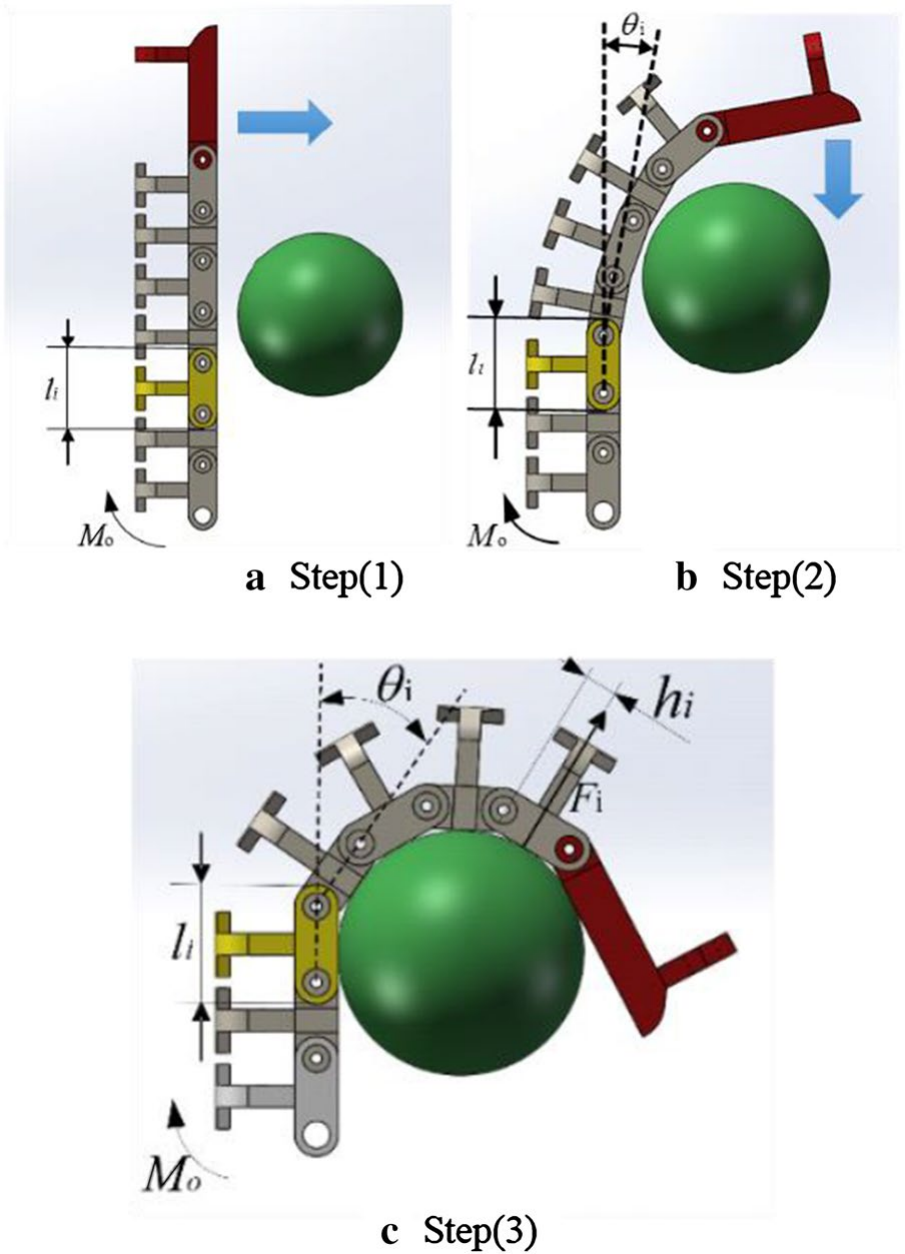
Correspondence of the physical quantities. **a** Step 1. **b** Step 2. **c** Step 3

The correspondence of the physical quantities is shown in Fig. 5.

For modularization of the joint units, we set:

1 $$l_{1} = l_{2} = \cdots = l_{i} = l_{i + 1}$$

2 $$\alpha_{i} = 0$$

3 $$d_{\text{i}} = 0$$

So we can get the transformation matrix between two adjacent joints, i.e.,

4 $${\mathbf{A}}_{i}^{i - 1} = \left[ {\begin{array}{*{20}l} {\cos \theta_{i} } \hfill & { - \sin \theta_{i} } \hfill & 0 \hfill & {l_{i} \cos \theta_{i} } \hfill \\ {\sin \theta_{i} } \hfill & {\cos \theta_{i} } \hfill & 0 \hfill & {l_{i} \sin \theta_{i} } \hfill \\ 0 \hfill & 0 \hfill & 1 \hfill & 0 \hfill \\ 0 \hfill & 0 \hfill & 0 \hfill & 1 \hfill \\ \end{array} } \right]$$

Then we can obtain the transformation matrix of joint coordinates $\{ i\}$ relative to the static coordinates $\left\{ 0 \right\}$:

5 $${\mathbf{T}}_{i}^{0} = {\mathbf{A}}_{1} {\mathbf{A}}_{2} \cdots {\mathbf{A}}_{i}$$

As ${\mathbf{A}}_{i}^{i - 1}$ (*i* = 1, 2… 15) is the function of the joint variable $\theta_{\text{i}}$, ${\mathbf{T}}_{i}^{0}$ is the function of the joint variable $\theta_{\text{i}}$. Therefore, one can figure out the position and posture of each joint by measuring the value of the joint variable $\theta_{i}$ (*i* = 1, 2… 15). And hence one can figure out the position and posture of the tentacle by measuring the value of the joint variable $\theta_{i}$ (*i* = 1, 2… 15).

### Grasping force analysis

In this paper, we replace rigid phalanx with artificial tentacles, hoping that the hand possesses high adaptability and grasping force. Therefore, the force analysis is essential and necessary. And we propose a mathematical model which is discussed in this section to analyze the distribution of grasping force. For simplification, the analysis neglects the gravity of the finger and the friction between joints and the object.

- $l_{i}$: the length of joint *i*, mm;
- $F_{i}$: the force at the contact point of joint *i* and object, *N*;
- $h_{i}$: the distance between the contact point of joint *i* and adjacent axes, mm;
- $M_{\text{o}}$: total output torque, Nm;
- $\theta_{i}$: angle of joint *i* and joint *i *− 1, °;
- $M_{i}$: the torque relative to the fixed point O which is caused by $F_{i}$, Nm.

According to mechanics’ law, the torque of force *F* with arm *L* is

6 $$M = F \cdot L$$

And according to Fig. 7, we can conclude that

7 $$\begin{aligned} M_{o} & = F_{o} \cdot h_{o} + F_{1} \cdot (h_{1} + l_{0} \cdot \cos (\theta )) \\ & \quad + F_{2} \cdot (h_{2} + l_{1} \cdot \cos (\theta_{2} ) + l_{0} \cdot \cos (\theta_{2} + \theta_{1} )) \\ & \quad + \cdots + F_{14} \cdot (h_{14} + l_{13} \cdot \cos (\theta_{14} ) + l_{12} \cdot \cos (\theta_{14} + \theta_{13} )) \\ & \quad + \cdots + l_{0} \cdot \cos (\theta_{14} + \theta_{13} + \cdots + \theta_{2} + \theta_{1} )) \\ \end{aligned}$$

We mark that

8 $$\begin{aligned} M_{i} & = F_{i} \cdot (h_{i} + l_{i - 1} \cdot \cos (\theta_{i} ) + l_{i - 2} \cdot \cos (\theta_{i} + \theta_{i - 1} ) \\ & \quad + \cdots + l_{0} \cdot \cos (\theta_{i} + \theta_{i - 1} + \cdots + \theta_{1} )) \\ \end{aligned}$$

Then, Eq. (7) can be expressed as:

9 $$\left\{ {\begin{array}{*{20}l} {M_{o} = \sum\limits_{i = 0}^{n} {M_{i} } } \hfill \\ {M_{i} = F_{i} \cdot (h_{i} + l_{i - 1} \cdot \cos (\theta_{i} ) + l_{i - 2} \cdot \cos (\theta_{i} + \theta_{i - 1} )} \hfill \\ {\quad + \cdots + l_{0} \cdot \cos (\theta_{i} + \theta_{i - 1} + \cdots + \theta_{1} )} \hfill \\ \end{array} } \right.$$

When we assume that each joint is small enough, we can consider the contact point between each joint and object to be the middle of the joint. In that case, the mathematical model can be simplified,

10 $$h_{i} + l_{i} /2$$

What’s more, we can consider every joint to be all the same, so it can be simplified as:

11 $$l_{\text{i}} = l_{\text{i-1}} = \cdots = l_{0}$$

So we finally get Eq. (12), i.e.,

12 $$\left\{ {\begin{array}{*{20}l} {M_{0} = \sum {M_{\text{i}} } } \hfill \\ {M_{\text{i}} = l_{\text{i}} F_{\text{i}} \left( {\frac{1}{2} + \cos (\theta_{\text{i}} ) + \cos (\theta_{\text{i}} + \theta_{\text{i-1}} )} \right.} \hfill \\ {\left. {\quad + \cdots + \cos (\theta_{\text{i}} + \theta_{\text{i-1}} + \cdots \theta_{1} )} \right)} \hfill \\ \end{array} } \right.$$

$\theta_{\text{k}}$ is always no larger than 180° and meanwhile no smaller than 0°. Then it can be concluded that:

1. $M_{i}$ decreases as $\theta_{i}$ increases when other variables are fixed.
2. $M_{i}$ decreases as $l_{i}$ decreases when other variables are fixed.

## Analysis results and discussion

**Fig. 6:**
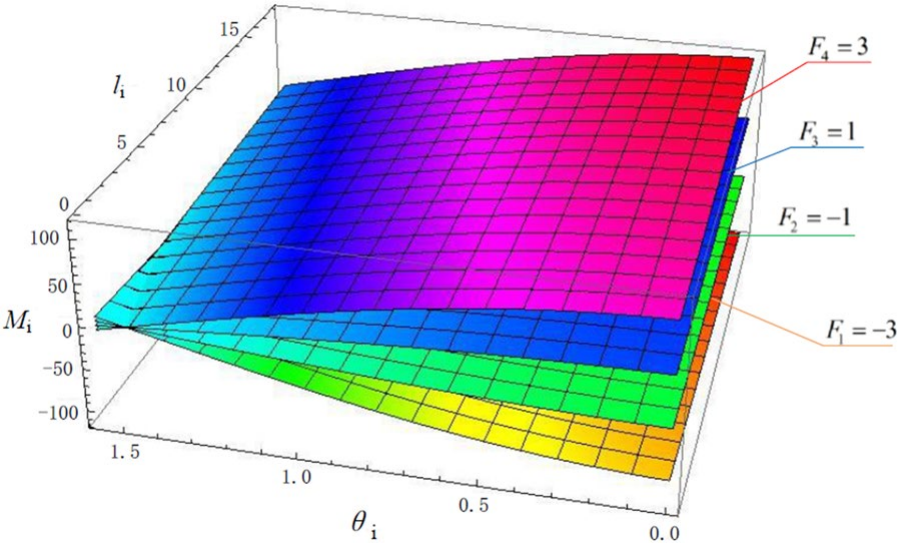
Relationship of $$M_{i} - l_{i} - \theta_{i}$$

**Fig. 7:**
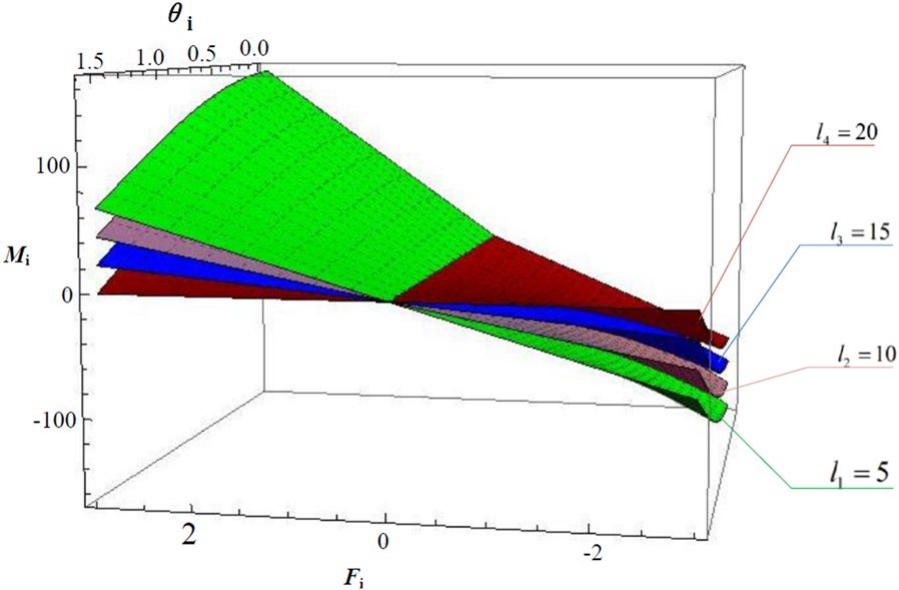
Relationship of $$M_{i} - F_{i} - \theta_{i}$$

**Fig. 8:**
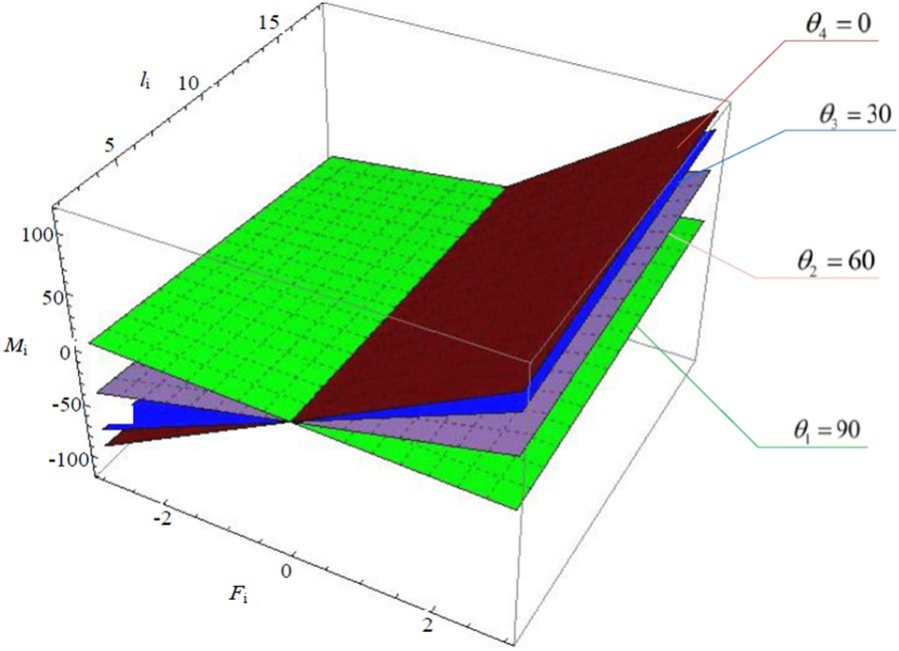
Relationship of $$M_{i} - l_{i} - F_{i}$$

We can draw images based on Eq. (12), which are shown in Figs. 6, 7 and 8.

The relationship of $M_{\text{i}} - l_{\text{i}} - \theta_{\text{i}}$ is shown in Fig. 6, where $F_{i}$ is − 3, − 1, 1 and 3. We can get the same conclusions from the figure:

1. $M_{i}$ decreases as $\theta_{i}$ increases when other variables are fixed.
2. $M_{i}$ decreases as $l_{i}$ decreases when other variables are fixed.

So we can draw a significant conclusion: Keeping $l_{i - 1}$, $l_{i - 2}$, …, $l_{0}$ fixed, when the grasping force and total output torque are identical, $M_{i}$ decreases as $l_{i}$ decreases, which demonstrates that the force distribution is more suitable for protecting object under this condition. That is to say, the OS Hand outperforms the traditional robotic hands in force distribution, and the OS Hand can better prevent object from being damaged.

And the relationship of $M_{i} - F_{i} - \theta_{i}$ is shown in Fig. 7, where $l_{i}$ is 5, 10, 15, 20.

The figure demonstrates that, with arbitrary length of joints, $M_{i}$ increases as $F_{i}$ increases and $M_{i}$ decreases as $\theta_{i}$ increases. However, $M_{i}$ decreases as $l_{i}$ decreases. Due to that, while choosing the parameters of the prototype, we should minimize the length of joint under the constraint of maximizing grasping force. That’s to say, the OS Hand performs better in protecting objects from being damaged.

The relationship of $M_{i} - l_{i} - F_{i}$ is shown in Fig. 8, and $\theta_{i}$ is 0°, 30°, 60° and 90°.

The figure demonstrates that, with arbitrary $\theta_{i}$, $M_{i}$ increases as $F_{i}$ increases and $M_{i}$ decreases as $l_{i}$ decreases. That is to say, the more flexible OS Hand can better protect object. Besides, grasping force has more influence on the torque $M_{i}$ than the length of joints. When the grasping force is assigned to −1 N, the influence on torque $M_{i}$ from the length of joint almost goes away. In order to fully unleash the advantages of OS Hand, input grasping force should be far from − 1 N.

## Experiment results and discussion

**Fig. 9:**
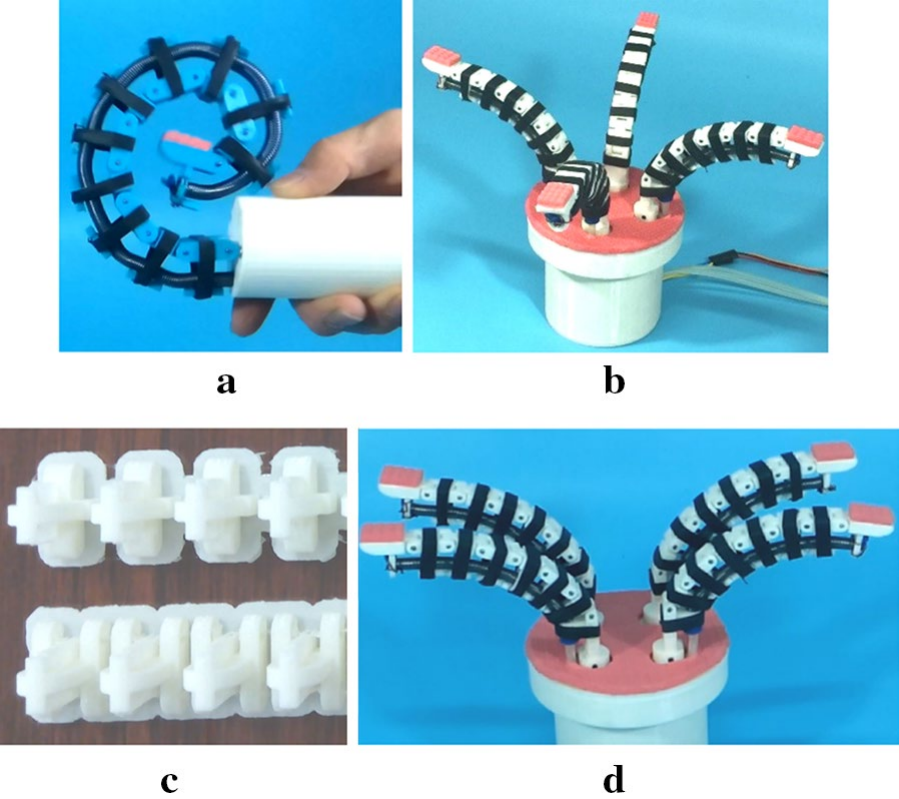
Prototype of the OS Hand. **a** Prototype of OS finger. **b** Prototype of OS Hand (gesture 1). **c** Parts needed to fabricate the OS finger. **d** Prototype of OS Hand (gesture 2)

To prove what we designed and analyzed are correct, grasping experiment of OS Hand was conducted. Figure 9a shows the prototype of the OS finger (in the closing pose of the finger for grasping objects). And the prototype of the OS Hand is shown in Fig. 9b, the artificial muscle is in initial state and the slight contraction of the artificial muscle makes the artificial tentacles open out. The parts needed to fabricate the OS Hand are shown in Fig. 9c. When the gear motor starts, the four meshed gears will rotate and the hand pose will change, as is shown in Fig. 9d.

**Fig. 10:**
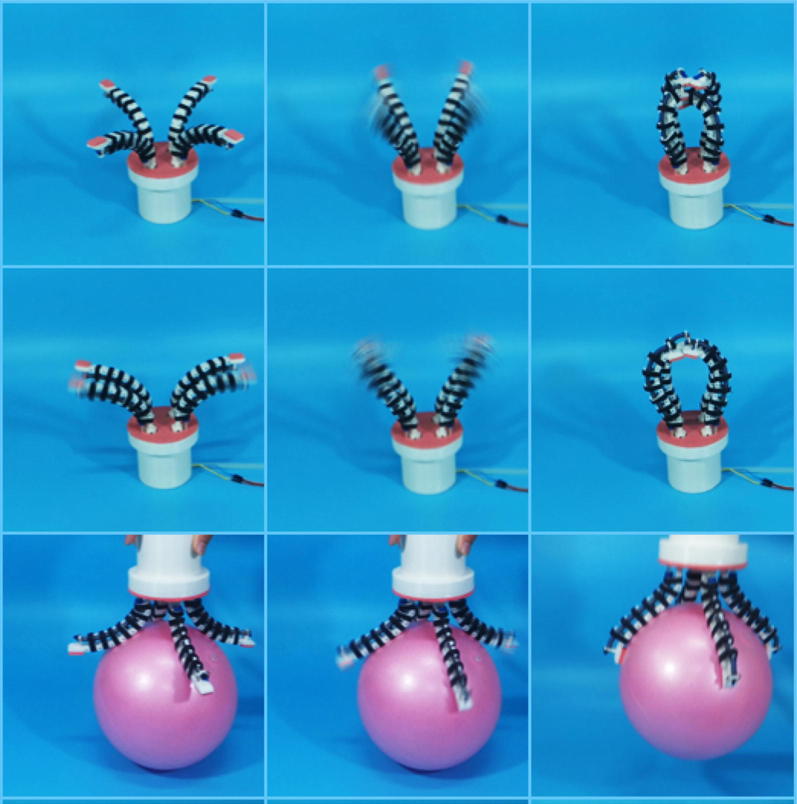
Grasping process experiment of OS Hand

Figure 10 shows the grasping progress. When starting the motor, the transmission plays a role and fluid is injected into the stretchable flexible tube, causing the elongation of the stretchable flexible tube, which drives the joints to rotate. The joints terminate until they contact object, and then, the process of adapting to object is finished. When releasing the object, fluid is extracted from the stretchable flexible tube, causing the contraction of the stretchable flexible tube. Then, the artificial tentacles open out and the object is released. It is worth noting that after the fluid is injected into the stretchable flexible tube, fluid cannot outflow spontaneously, which provides reliable grasping.

**Fig. 11:**
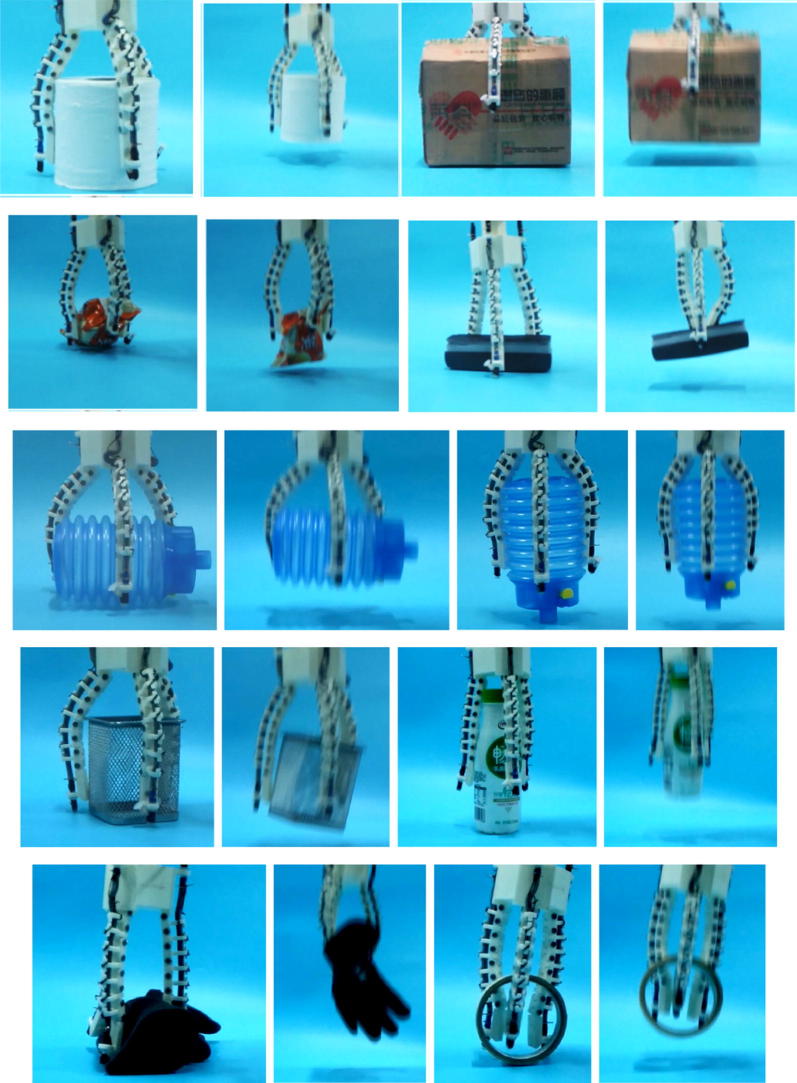
Experiments of OS Hand with three tentacles

**Fig. 12:**
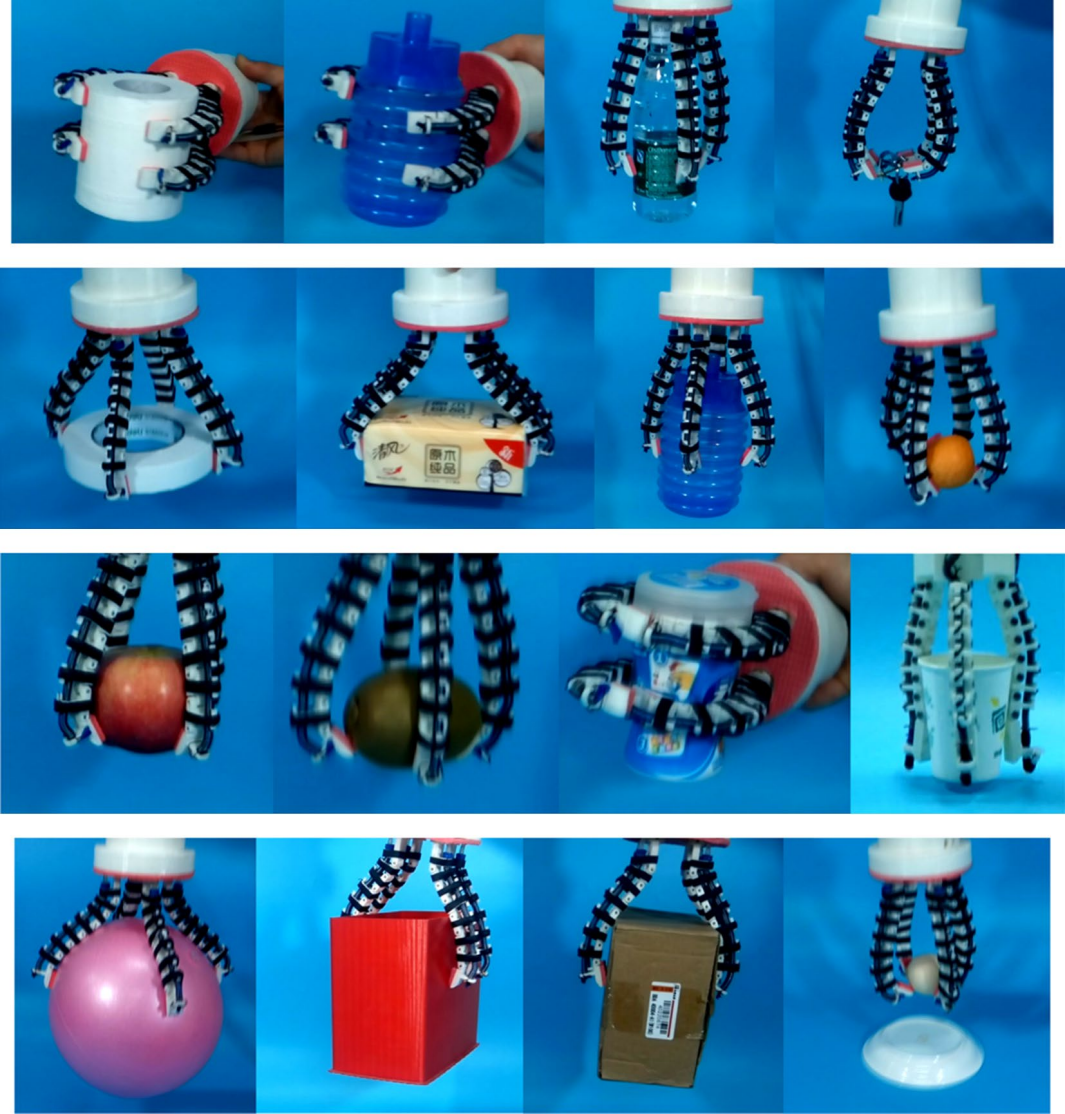
Experiments of OS Hand with for tentacles

**Fig. 13:**
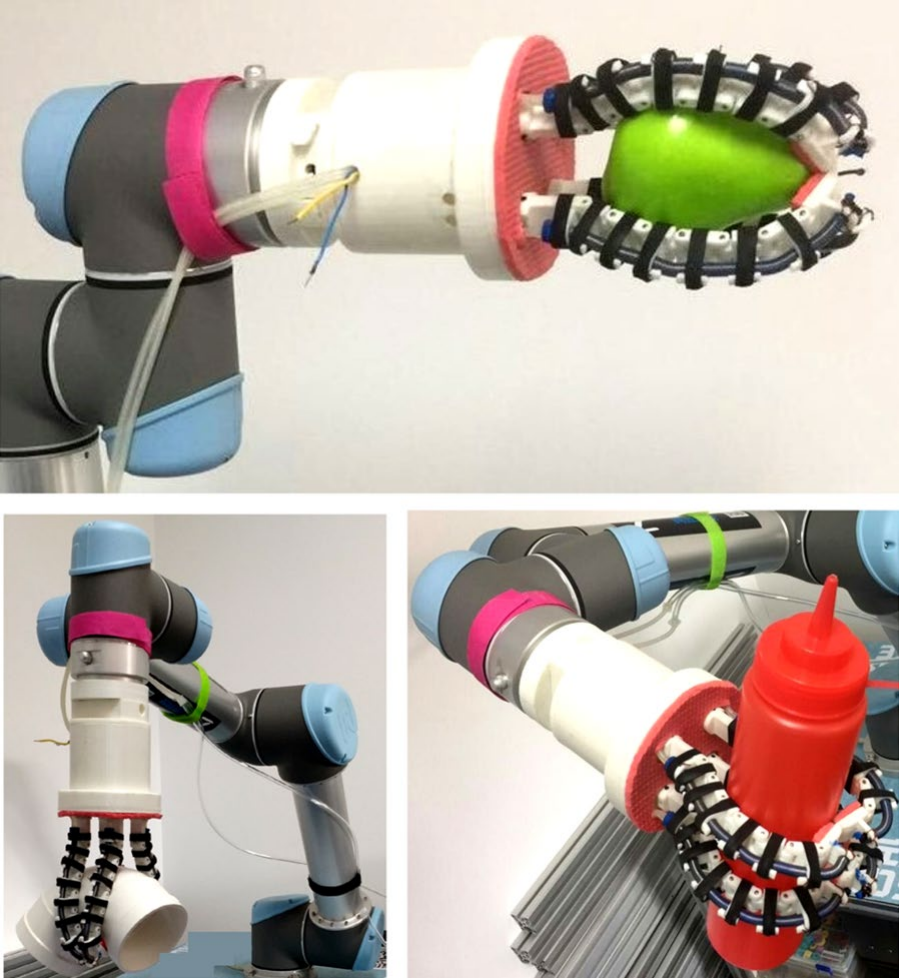
Experiments of OS Hand connected with robot arm

Furthermore, in order to test the grasping performance of the OS Hand, various kinds of objects are adopted in the experiments, as is shown in Figs. 11, 12 and 13.

The figures show that the OS Hand combines good qualities of both rigid grasp of traditional grippers and form-fitting grasp of flexible hands. The OS Hand can match the shape and size of the object automatically. Experimental results confirm that the OS Hand is valid for precise pinching, self-adaptive powerful encompassing, proving its practicability.

In addition, during the experiment we have also measured the size and weight of the grasped object to verify its practicability. In fact, the maximum grasping weight of the robot hand has a lot to do with the motor and the stretchable flexible tube. And under our experimental conditions, a bunch of keys, an egg, a ball with a radius of 120 mm, a box of 300 mm × 150 mm × 260 mm, a whole bottle of 350 ml of water, etc., can all be grasped steadily. We also use a dynamometer to measure the maximum grasping weight of the prototype and that is about 6.1 N. We believe that the maximum grasping weight will be much greater with better materials and manufacturing.

In order to test the timeliness, we also attach great importance to the response time of OS Hand. We test the closing time of the hand for grasping objects and its opening time for releasing objects. Subject to the speed of our motor, the response time is between 1.2 and 4.7 s. Considering that pneumatics power drive will respond much more rapidly, we connect the OS Hand with a robot arm and drive the hand with pneumatics power and test the response time, as is shown in Fig. 13. And the experimental results are very exciting. The hand is almost instantaneously grasping and releasing, and the response time is less than 1 s.

From the experiment result, we can find the advantages of OS Hand as follows:

- Great performance of underactuation and self-adaptation: In the experiment, there are even as many as 32 degrees of freedom in the OS Hand, but only two motors are enough to actuate it. The redundancy of the hand is high, and the adaptability is great;
- High integration: modular design is adopted in the design of the hand. The motor, the transmission, the fluid and the control circuit all can be embedded in the palm;
- Strong grasping ability: the joints of the eight serial-hinged joints are made of rigid links, providing support for artificial tentacles and improving the grasping strength. The strength of joints and stretchable flexible tube are adequate to sustain heavy objects. Reasonable structure design and stretchable flexible tube realize the stable and high adaptive grasping. The hand can achieve the encompassing grasp and fingertip grasp. All kinds of common objects can be grasped steadily.

## Conclusions

Inspired from flexible bending of octopus’ tentacles and outside-driving kind of traditional hand exoskeletons, this paper proposes a novel self-adaptive underactuated multi-fingered hand (OS Hand), which has four flexible tentacles.

1. Each tentacle can execute different grasping modes depending on the shapes and dimensions of the objects grasped and grip objects in a gentle and form-fitting manner.
2. The OS Hand combines good qualities of both powerful grasp of traditional grippers and form-fitting grasp of flexible hands.
3. Experimental results show that the OS Hand is valid for precise pinching, self-adaptive powerful encompassing, and grasping forces that are freely changeable in a wide range.
4. With the advantages of high self-adaptation, various grasp configurations and large range of grasping forces, the OS Hand has a wide range of applications in the area of service robotics which requires a lot of flexible operations of general grasping, moving and releasing.
